# Reply to Keeler, J.L.; Steinhäuser, J.L. Comment on “Wu et al. Peripheral Biomarkers of Anorexia Nervosa: A Meta-Analysis. *Nutrients* 2024, *16*, 2095”

**DOI:** 10.3390/nu17172874

**Published:** 2025-09-05

**Authors:** Ya-Ke Wu, Hunna J. Watson, Aaron C. Del Re, Jody E. Finch, Sabrina L. Hardin, Alexis S. Dumain, Kimberly A. Brownley, Jessica H. Baker

**Affiliations:** 1 School of Nursing, University of North Carolina at Chapel Hill, Chapel Hill, NC 27599, USA; 2 Department of Psychiatry, University of North Carolina at Chapel Hill, Chapel Hill, NC 27599, USA; hunna_watson@med.unc.edu (H.J.W.); kim_brownley@med.unc.edu (K.A.B.); 3 School of Psychology, Curtin University, Bentley, WA 6102, Australia; 4 School of Paediatrics, Division of Medicine, The University of Western Australia, Crawley, WA 6009, Australia; 5 Del Re Data & Statistical Consulting, San Diego, CA 91910, USA; stats@acdelre.com; 6 Department of Psychology, University of Kassel, 34127 Kassel, Germany; 7 Department of Psychology, Georgia State University, Atlanta, GA 30302, USA; jodyfinch36@gmail.com; 8 National Center for PTSD, VA Boston Healthcare System, Boston, MA 02130, USA; sabrinah97@gmail.com; 9 Department of Psychology and Neuroscience, University of North Carolina at Chapel Hill, Chapel Hill, NC 27599, USA; alexissd@live.unc.edu; 10 Equip Health, Inc., P.O. Box 131747, Carlsbad, CA 92013, USA; bakerjh10@gmail.com

Thank you for the constructive feedback on our recent meta-analysis on peripheral biomarkers in anorexia nervosa (AN) [1,2]. We appreciate your close reading of our work and your constructive engagement with the data.

We recognize that conducting a meta-analysis of this scale—incorporating data on 52 distinct peripheral biomarkers across a diverse population—is inherently complex and subject to inevitable limitations, and we welcome all feedback that helps strengthen the validity and reliability of our findings. We fully acknowledge the importance of accurate data inclusion and are grateful for your observation regarding the unintentional duplicate entry of the study by Eddy et al. [3] in the forest plot for brain-derived neurotrophic factor (BDNF). We agree that, in line with Cochrane guidance, the unintentional duplication of studies may introduce bias and potentially impact the pooled effect size.

Upon examination of the methodological concerns noted by the commentators, we recalculated g for BDNF, excluding the duplicate entry of Eddy et al. [3]. The updated estimate was g = −0.34 (95% confidence interval [CI]: −0.82, 0.15), *p* = 0.08, compared with our original result of g = −0.22 (95% CI: −0.61, 0.18), *p* = 0.07. We note that the commentators also conducted a re-analysis incorporating two additional studies meeting the inclusion criteria that were inadvertently and regrettably not included in our database during the initial search phase. We have no reason to doubt the accuracy of the result they reported: g = −0.59 (95% CI: −1.02, −0.16). This updated effect size suggests a more robust difference in BDNF levels between individuals with AN and controls than our original analysis indicated. We believe these corrections are important for readers to consider when interpreting the updated result for BDNF.

We appreciate your observation regarding the possible omission of additional eligible studies on BDNF, C-reactive protein, and lymphocytes. While we carefully followed our predefined inclusion and exclusion criteria, the possibility remains that some eligible studies were inadvertently missed during our initial screening of over 2000 articles. Any such omission was unintentional, and we acknowledge this as a limitation of our review. Unfortunately, due to resource constraints, we are currently unable to conduct another full review of the literature. However, as new evidence on potential biomarkers for AN continues to emerge rapidly, we encourage other researchers to take on this important and evolving challenge.

We fully recognize that identifying reliable biomarkers for AN is not the task of a single research team, but rather a shared responsibility of the entire scientific community. The biological underpinnings of AN are multifaceted, and it is impossible for any single study—or even a single meta-analysis—to capture the full picture. Strengthening the evidence base requires collective effort: from study design and data sharing, to replication, rigorous synthesis, and peer review. Only through collaboration can we build a truly comprehensive and reliable understanding of the molecular mechanisms at play, and ultimately, improve the lives of individuals affected by this severe disorder.

The identification of reliable biomarkers is indeed a key step toward the development of personalized treatments for AN. We hope readers can appreciate the significant effort our team has invested in conducting this large-scale meta-analysis, synthesizing findings across multiple peripheral biomarkers. This work represents a major step toward systematizing what is known, highlighting areas of consistency as well as gaps in the literature. However, we also view it as a foundation—an invitation for further refinement, expansion, and innovation by others in the field. We are encouraged by the engagement of the research community in this shared goal and are committed to maintaining methodological transparency and rigor. At the same time, we hope to inspire the next generation of researchers to take on this important challenge—to build on this work and continue pushing the boundaries of our understanding to develop more effective interventions for individuals with AN.
